# Investigation of the THOR Anthropomorphic Test Device for Predicting Occupant Injuries during Spacecraft Launch Aborts and Landing

**DOI:** 10.3389/fbioe.2014.00004

**Published:** 2014-03-17

**Authors:** Jeffrey T. Somers, Nathaniel Newby, Charles Lawrence, Richard DeWeese, David Moorcroft, Shean Phelps

**Affiliations:** ^1^Science Technology and Engineering Group, Wyle, Houston, TX, USA; ^2^Glenn Research Center, National Aeronautics and Space Administration, Cleveland, OH, USA; ^3^Civil Aerospace Medical Institute, Federal Aviation Administration, Oklahoma City, OK, USA; ^4^Georgia Tech Research Institute, Atlanta, GA, USA; ^5^Georgia Institute of Technology, Atlanta, GA, USA

**Keywords:** injury criteria, spaceflight, dynamic loads, anthropometric test device, test device for human occupant restraint, injury assessment reference values

## Abstract

The objective of this study was to investigate new methods for predicting injury from expected spaceflight dynamic loads by leveraging a broader range of available information in injury biomechanics. Although all spacecraft designs were considered, the primary focus was the National Aeronautics and Space Administration Orion capsule, as the authors have the most knowledge and experience related to this design. The team defined a list of critical injuries and selected the THOR anthropomorphic test device as the basis for new standards and requirements. In addition, the team down-selected the list of available injury metrics to the following: head injury criteria 15, kinematic brain rotational injury criteria, neck axial tension and compression force, maximum chest deflection, lateral shoulder force and displacement, acetabular lateral force, thoracic spine axial compression force, ankle moments, and average distal forearm speed limits. The team felt that these metrics capture all of the injuries that might be expected by a seated crewmember during vehicle aborts and landings. Using previously determined injury risk levels for nominal and off-nominal landings, appropriate injury assessment reference values (IARVs) were defined for each metric. Musculoskeletal deconditioning due to exposure to reduced gravity over time can affect injury risk during landing; therefore a deconditioning factor was applied to all IARVs. Although there are appropriate injury data for each anatomical region of interest, additional research is needed for several metrics to improve the confidence score.

## Introduction

### Purpose

The objective of this work was to: (1) identify a list of critical spaceflight injuries from dynamic loading that need to be protected against to enable mission success, (2) identify an anthropomorphic test device (ATD) to be used to predict the threshold at which human injuries will occur, and (3) develop a table of ATD thresholds known as injury assessment reference values (IARVs) for each critical injury. The eventual goal is to develop a standardized test methodology (i.e., ATD, seat, suit, acceleration profiles, etc.) for inclusion in National Aeronautics and Space Administration (NASA) Standard 3001, and all related program requirements (National Aeronautics and Space Administration, 2011). The information included is based on available data at the time of the report and upon opinions from experts at NASA, the National Highway Traffic Safety Administration (NHTSA), and the Federal Aviation Administration (FAA). The team considered occupant protection (OP) needs during dynamic phases of spaceflight, which include abort (pad abort and ascent abort) as well as re-entry and landing. Although various spacecraft designs were considered, the primary focus was the NASA Orion capsule, as the authors have the most knowledge and experience related to this design.

### Spaceflight design considerations

#### Vehicle designs

Reaching space requires an extreme amount of kinetic energy, and effective systems to dissipate this energy on the return to Earth. While most of this energy is controlled, dissipated, or absorbed by the vehicle, some amount of kinetic energy may be transmitted to the occupants aboard the spacecraft. This energy, if not properly managed, may cause injury to the crewmembers. Vehicle design is an important consideration for managing this energy, particularly during launch aborts and landing.

##### Launch and abort systems

Currently, chemical rockets are used to launch humans into space. These systems typically accelerate the crew vehicle to orbital velocities >6,900 m/s (15,430 mph) to attain low earth orbit (LEO) within 10 min of launch. These sustained accelerations are designed to be well within human tolerance. Because of the amount of energy stored in the launch vehicle (either liquid or solid propellant), there are failure modes that necessitate the design of abort systems.

Most human spaceflight vehicles designed to date have included launch phase abort capabilities. For the Mercury and Apollo programs in the U.S. – as well as the Soyuz program in Russia – a launch escape system was included in the spacecraft design to allow quick separation of the crew module away from the main vehicle in case of a catastrophic failure of the launch system.

For the Russian Vostok and Buran programs, as well as the U.S. Gemini program, ejection seats were included in the spacecraft design to allow crewmembers to escape separately from the entire launch vehicle, although they could only be operated during a very short period of the launch profile. No abort capabilities existed outside of this period until sufficient altitude was reached to allow for a normal separation and descent. The U.S. Space Transportation System Program (the formal name for NASA’s “Space Shuttle” program) included four primary elements: an orbiter spacecraft (Space Shuttle), two solid rocket boosters (SRB), an external tank housing fuel and oxidizer, and the three Space Shuttle main engines. The Space Shuttle included ejection seats that were disabled after the first four flights and eventually removed (Jenkins, 1999).

All future NASA vehicles are required to have a crew escape system (National Aeronautics and Space Administration, 2012a). Because crew escape systems must quickly separate the crew away from the launch vehicle, the crew may be exposed to high dynamic loads. These loads will vary depending on vehicle design, phase of flight, and other vehicle performance characteristics.

##### Capsule landing systems

**Figure 1:**
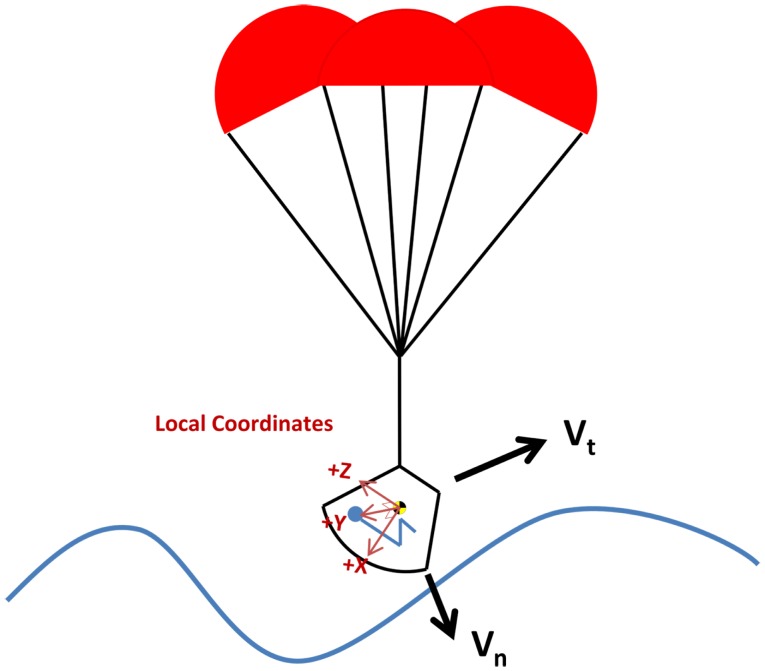
**Nominal capsule landing orientation with respect to water (land landing similar)**.

The capsule design was the original design chosen for human spaceflight primarily because it is mass efficient and simple. To date, this design has been used for all human spaceflight programs, with the exception of the U.S. Space Shuttle. Capsule vehicles typically land with the crew in a seated positioned reclined 90° from vertical on their back, contacting the impact surface feet first, resulting in a combined +*X* (eyeballs in) and +*Z* (eyeballs down) primary landing load (see Figure 1), although landing dynamics are heavily dependent on the specific design and failure modes.

The Mercury, Apollo, multi-purpose crewed vehicle (MPCV), and SpaceX Dragon are designed to land primarily in the ocean, but can also land on land in contingency cases. The Vostok, Voskhod, Soyuz, and Boeing CCT-100 are all designed for primary landing on land, with contingency landing capability in water. Capsules may include additional energy management features such as retrorockets, airbags, energy-absorbing structures, and/or stroking seats.

##### Lifting body design

A lifting body design improves upon the capsule design by adding lifting surfaces to increase maneuverability and cross-range performance. Sierra Nevada’s Dream Chaser is an example of a lifting body design, and is based on NASA’s HL-20 prototype (National Aeronautics and Space Administration, 2013; Sierra Nevada Corporation, 2013). From an OP standpoint, a lifting body represents a less violent landing environment, as it does not rely solely on parachutes to dissipate landing velocity. Even in off-nominal landing conditions, this design feature is expected to reduce the potential for injury.

#### Landing loads and vectors

Because relatively little is known about landing load magnitudes and direction vectors for future commercial vehicles (SpaceX Dragon, Boeing CCT-100, and Sierra Nevada Dream Chaser), knowledge of the MPCV design was used as a logical basis for this work. This is likely the bounding case for all of the commercial vehicles for several reasons. First, the MPCV is larger and heavier than its commercial counterparts, which should result in larger landing loads than the commercial vehicles. Second, the MPCV was evaluated for its performance during land landings. Although the landings were severe, they were not significantly worse than some off-nominal water landing scenarios. In addition, the MPCV was not designed for a primarily land landing, so vehicles that are designed primarily for land landing (like the CCT-100) would likely have a softer land landing. Finally, for the lifting body designs, the MPCV water impact will be more severe than off-nominal landings encountered during a horizontal landing.

For the MPCV, the landing loads are primarily +*X* (eyeballs in) and +*Z* (eyeballs down). This is a similar orientation to the Apollo CM (see Figure 1). The +*X* component is primarily due to vertical velocity of the vehicle and is dependent on parachute performance. In the case of a two-parachute landing, the vertical velocity will be higher, resulting in a higher +*X* load. The +*Z* component is driven by two factors. First, the horizontal wind and wave speed affects the horizontal velocity. At high wind speeds the +*Z* load is much higher. Second, the vehicle is designed with a set hang angle, tilting the entire capsule so that only the edge of vehicle contacts the water first. Even with no horizontal wind, this hang angle will impart a +*Z* axis load based on the vertical velocity. In addition, wave slope during water landings and terrain slope during land landings contribute to the effective impact angle.

Although these vectors are primary to the landing load, the load is also dependent on the vehicle maintaining the correct orientation, so that the crew are oriented correctly at landing (i.e., feet first). An onboard control system is needed to maintain this orientation. Without a control system, the vehicle can rotate before impact, imparting loads in other directions. The landing loads may be quite complex, necessitating consideration of all vehicle dynamics.

#### Landing modes

Each spaceflight vehicle design has a unique launch, abort, re-entry, and landing environment. Each vehicle is optimized for its particular “nominal” case landing, or the landing that has the highest probability of occurring. To determine this landing mode, detailed analyses of the vehicle systems and environmental factors are conducted. These analyses identify the distribution of all possible landings related to the normal and tangential velocities. Assuming a normal distribution of all landing probabilities, thresholds for nominal and off-nominal can be defined (see Table 1). It should be noted that for capsule-based vehicles, even nominal landing dynamics are more like an automobile accident, than normal automotive accelerations.

**Table 1:** **Comparison of possible nominal and off-nominal distribution thresholds**.

Threshold	Percentage of landing cases	Percentage of cases outside of range	Exp. freq. outside of range	Approx. occurrence for one landing outside of range (assuming four flights per year)	Approx. design levels for Orion
≤μ + 1σ	84.1	15.9	1 in 6	Every 18 months	
≤μ + 1.5σ	93.3	6.7	1 in 15	Every 4 years	Nominal
≤μ + 2σ	97.7	2.3	1 in 44	Every 11 years	
≤μ + 2.5σ	99.4	0.6	1 in 161	Every 40 years	Off-nominal
≤μ + 3σ	99.9	0.14	1 in 769	Every 200 years	

Although Orion used a slightly different method of defining nominal and off-nominal, based on the probabilities of off-nominal landings and the associated levels of risk for each, the total landing cases that are ≤μ + 1.5σ threshold are an approximation of the Orion risk level for nominal, and the landing cases between μ + 1.5σ and μ + 2.5σ are similar to the risk level for off-nominal. All cases above μ + 2.5σ landing case would be contingency and not considered for design purposes (0.6% of cases). Using this definition, an off-nominal event will occur approximately every 4 years and a contingency event every 40 years, or effectively never (assuming four launches per year).

#### Crew deconditioning

Several physiologic changes occur in response to microgravity. The two main changes concerning impact tolerance are bone mineral density loss and muscular tissue atrophy.

During prolonged spaceflight, skeletal density changes primarily in the lower extremities and spine (Lang et al., 2004) consistent with Wolff’s law (Wolff, 1986). Studies conducted using dual energy X-ray absorptiometry (DXA) have shown bone mineral density decreases on average of 1–1.6% in the spine, femoral neck, trochanter, and pelvis, with an average loss of 1.7% in the tibia after only 1 month in microgravity (LeBlanc et al., 1998; Vico et al., 2000). Because skeletal deconditioning is time dependent, any method for accommodating the losses will be mission length specific.

Changes in muscle mass and strength also occur, and are dependent on the exercise regime employed during spaceflight. During Skylab missions, leg volume decreased by 7–10% (Thornton and Rummel, 1977) and up to 19% in crewmembers aboard the Mir space station (Stein, 1999; LeBlanc et al., 2000). The muscle loss experienced by crewmembers is also selective; muscle fiber size in the vastus lateralis decreased after 5–11 days in flight at different rates. Edgerton et al. (1995) and Zhou et al. (1995) found decreases of 16% in Type I, 23% in Type IIa, and 36% in Type IIb fibers. In terms of muscle strength during 6 month missions, astronauts experienced muscle strength loss up to 24% at the knee and up to 22% in the ankle (Gopalakrishnan et al., 2010).

It is currently unclear how these changes affect human physiology and impact tolerance in the setting of spaceflight and landing following long duration mission profiles. However, it is fairly evident that greater OP measures will be needed for deconditioned crewmembers. Currently, these effects are accounted for by applying lower dynamic load limits, which are based on NASA’s Integrated Medical Model (Lewandowski et al., 2008). The Integrated Medical Model of bone loss is derived from bone mineral density changes, not on actual measured and scientifically validated human impact tolerance in these conditions. This approach may be acceptable for short stays on the international space station (ISS), but may not apply to or protect against the deleterious physiological effects of longer-duration missions to near-Earth objects, the moon, and/or Mars, since there is little known about spaceflight deconditioning beyond 6 months to 1 year. In addition, inflight countermeasures are being developed to counteract these physiological changes, so in the future, lowering the response limits may not be necessary.

#### Injury risk posture

To gain insight into what NASA’s injury risk posture should be, it is helpful to review other industries and their respective risk postures derived from their contextual operational scenarios based upon scientific evidence gathered therein.

For the automotive industry, specifically passenger cars, most injury limits are based on a 5–50% risk of an abbreviated injury scale (AIS) 3+ injury, which delineates the occurrence of a severe injury (Association for the Advancement of Automotive Medicine, 2005). Although this seems like an objectionable risk posture, there are two main reasons that this posture is acceptable for the automotive industry. First, these limits are based on standardized tests that represent a worst case scenario, and not a “representative” collision. Second, the overall probability of a person being in a crash any time he or she gets into a vehicle is very remote (1 in 120,000) for passenger vehicle usage (National Highway Traffic Safety Administration, 2007, 2009). Therefore, after considering the severity of the collision and the probability of actually having a collision, the total risk of injury to automobile drivers is very low.

For military aircraft, the situation is similar, although more inherent risk is involved. Military aircraft (both fixed and rotary wing) are designed to allow for a higher risk posture than what is desired and acceptable for spacecraft occupants assigned to NASA. Again, these higher levels of risk are considered acceptable given that the overall risk of injury per “sortie” (defined as one deployment or dispatch – launch with subsequent landing – of a military aircraft on a mission) is 1 in 670 (or less), even though this is significantly higher than the risk seen in the operation of passenger cars (Mapes, 2006).

For NASA, the situation is very different. With passenger vehicles, millions of miles are driven each year with a relatively low risk of collision or injury. For the most part, passenger vehicle risk is constant during the entire trip or “sortie.” Similarly, for military aircraft, thousands of flight hours are logged with relatively low risks of injury, and like passenger vehicles, there is significant risk during the entire mission although this risk is a direct result of different variable causes (enemy fire, mechanical failures, weather, pilot errors, human factors, etc.). However, unlike passenger vehicles, military aircrews are subject to a higher risk of injury during takeoff and landing. This risk is closer to NASA’s environment where risk of injury due to dynamic loads is concentrated during launch and landing phases of operations when flight produces the highest loads on the vehicle. Unlike passenger vehicles and military aircraft, there is very low risk of injury due to impact once a stable orbit has been reached, due primarily to the fact that there are very small loads applied to the vehicle structure during this phase of flight. Additionally, the launch, launch abort, and landing environments for NASA are extreme in nature compared with what is nominally experienced during automobile driving or civilian/military flight. The differences between automotive, military, and NASA operational environments make it important to consider the *overall acceptable probability of injury*, given that occupants in a space vehicle will incur impact conditions every time the vehicle returns to Earth.

#### Suit considerations

One of the unique aspects of the NASA environment is the pressure or space suit. This suit protects the crew from the vacuum of space by providing a pressurized environment around the body, a breathable atmosphere, thermal protection, and micrometeorite protection (when outside the vehicle). In addition to these basic functions, other considerations in suit design include mobility, fit on a wide range of crewmembers, and contingency extravehicular activity (EVA). With all of these demands on the suit, the final design is often not optimized for OP. There are several considerations for the occupant during abort and landings that relate to suit design. First, the suit, unlike most clothing, may contain rigid elements. Depending on the placement of these elements, point-loads or blunt trauma may occur resulting in crew injury (McFarland and Dub, 2010). Therefore, the placement and design of these components are critical to protecting the crew (Danelson et al., 2011). Second, head-mounted mass can pose a serious threat to crewmembers if the additional mass is carried by the neck (Radford et al., 2011). Finally, because the suit is a pressure garment, there is a chance of landing with the suit inflated. In this case, the vehicle restraint system is no longer restraining the crewmember, but is instead restraining the suit. Inside the suit, the crewmember may be free to move, increasing the possibility of injury. In all, the suit presents a unique challenge that must be addressed to prevent crew injury.

#### Gender and anthropometrics

Unlike previous NASA capsule designs, future NASA vehicles must be capable of accommodating men and women in a wide range of anthropometrics. Current NASA vehicle requirements stipulate a vehicle must accommodate a 1st percentile female to a 99th percentile male. Protecting for such a wide range of sizes is a challenge. Most OP data are based either on young, male, military subjects, or on elderly male post-mortem human surrogates (PMHS). As of 2011, the astronaut corps median age is 47.1 years for males (range 35–56) and 43.3 years for females (range 32–52), with males accounting for 76% of the corps. In terms of anthropometry, stature is 177.3 ± 4.9 cm (4th–95th percentile) for male crewmembers, and 168.9 ± 4.3 cm (25th–97th percentile) for female crewmembers. For weight, male crewmembers are 79.3 ± 6.9 kg (6th–75th percentile), and 63.2 ± 8.9 kg (4th–65th percentile) (as shown in Table 2). Because of the wide range of demographics, accurately determining injury risk for the entire range within the astronaut corps is difficult, and the results will contain a certain amount of uncertainty.

**Table 2:** **NASA astronaut corps, gender, and anthropometric distribution (as of 2011)**.

Gender	Median age (range)	Percentage of corps	Stature (percentile)	Weight (percentile)
Male	47.1 (35–56)	76	177.3 ± 4.9 cm (4th–95th)	79.3 ± 6.9 kg (6th–75th)
Female	43.3 (32–52)	24	168.9 ± 4.3 cm (25th–97th)	63.2 ± 8.9 kg (4th–95th)

### Current NASA standards

Currently, NASA standards for transient accelerations (≤0.5 s) are based primarily on the Brinkley dynamic response criterion (BDRC) (National Aeronautics and Space Administration, 2011). The BDRC is a simple, lumped-parameter, and single degree of freedom model that estimates the whole body response due to applied acceleration, and is computationally efficient and requires very little in terms of validation testing.

However, the BDRC has limitations (Somers et al., 2013). It only predicts ranges of injury risk and cannot provide information as to the severity or anatomical location of an injury. Only the +*Z* axis injury risk is well validated (with operational ejection seat data); the ±*X*, ±*Y*, and −*Z* injury risk levels are statistically limited (Brinkley, 1985). In addition, these data do not account for the interactions between crewmembers, suit, and seat. The seat and helmet used in the human testing and development of the BDRC are very different from what is actually planned for MPCV. Previously performed tests did not typically include a suit, so suit interactions are not accounted for in the injury risk prediction.

Given the limitations of the current NASA Standards, the human research program (HRP) and the NASA Engineering and Safety Center (NESC) began studying alternatives for inclusion in the standard. This standard update work is primarily focused on the Orion vehicle.

### Expert panel summit

After working with the Orion design exclusively, the OP team held an expert summit in Houston in June 2010. The goal of the summit was to develop an OP plan that would not only further the Orion effort, but also adapt to the commercial crew vehicles.

Experts from the U.S. Army, U.S. Navy, U.S. Air Force, FAA, NHTSA, Indy racing league (IRL) (INDYCAR™), university researchers from Virginia Tech, Wake Forest University and Wayne State University, and automotive biomechanics experts attended the summit. NASA personnel from several areas including the Human Health and Performance Directorate (HRP, the Biomedical Research and Environmental Sciences Division, and the Space and Clinical Operations Division), the Astronaut Office, the Safety and Mission Assurance Directorate, and the NESC also participated.

Group consensus was reached on the forward plan consisting of the following elements:

- Phase 1 – literature review and standards framework.
- Phase 2 – ATD testing and finite element model (FEM) assessment.
- Phase 3 – human exposure data mining.
- Phase 4 – human testing and correlation to ATD response.
- Phase 5 – injury risk functions and NASA standards development.

Only Phase 1 of the forward plan is discussed in this report.

## Literature Review and Standards Framework Development

Based on the results of the expert panel summit, work began to determine a list of critical injuries that need to be mitigated, the best ATD for predicting biodynamic responses, and IARV needed to mitigate the injury risk. Based on current literature, a framework was developed for completing these tasks and identifying areas in need of additional research.

### Critical injury definition

The expert panel identified the need for a concise list of “critical” injuries that NASA would need to mitigate. Entries could be based on likelihood (based on previous spaceflight experience) or those that could cause the crew to be incapable of performing post flight tasks. The list is not all-inclusive, but is intended to protect the crew from injuries that if successful, would also allow a level of protection from more severe injuries. For example, if NASA were to protect against rib fractures and lung contusions, the assumption is that other internal organs would be protected to the same level as well.

The list of injuries was used to form the basis of the ATD selection. The ATD needed to be capable of generating a response that is relevant to the types of injuries NASA wishes to mitigate. The injury list combined with the selected ATD would ultimately drive the selection of the injury metrics and IARV.

Before establishing a critical injury list, several assumptions were made based on the existing NASA standards as follows:

- A five-point (or better) racing harness would be used.
- Transient accelerations (<0.5 s) would not exceed a moderate injury risk levels.
- Sustained (>5 s) linear accelerations, rotational accelerations, rotational velocities, and acceleration rate of change would not exceed levels specified in the standard (NASA Standard 3001) (National Aeronautics and Space Administration, 2011).
- Transient rotational accelerations would not exceed levels specified in the standard (NASA Standard 3001) (National Aeronautics and Space Administration, 2011).
- Minimal or no body movement (based on the Brinkley Amplification Rule).
- The vehicle would maintain an occupant survivable volume.
- Requirements would be met as for any other vehicle such as sharp edges, pinch points, etc.

Some assumptions were developed that were thought to be general enough to encompass most future NASA vehicles, yet constrained enough to allow a useful set of injuries to be defined. These are:

- Crewmembers may be in a pressurized suit, an unpressurized suit, or unsuited.
- The crew would be recovered within 24 h (this is based on analyses conducted during NASA’s Constellation Program showing that most locations worldwide can be reached within this timeframe).
- The crew must be protected to the extent that all post-landing egress tasks can be completed.
- Crewmember tasks required to egress the vehicle post-landing are assumed to be similar in human performance to Orion (i.e., similar physical abilities needed, but not necessarily the same tasks).
- Dynamic loads experienced by the crew would be less than or equal to the current predictions for the Orion vehicle (within the same order of magnitude of the current Orion assumed loads).
- Design of the vehicle would prevent inadvertent contact with vehicle interior excluding the seat and suit (i.e., seat can stroke into other structures, knees can’t contact control panel, and stowed items will not be free).
- Only considering injuries induced by dynamic loads (i.e., not considering inhalation dangers, fire, etc.).

Based on these assumptions, the team identified the critical injuries in several regions including the head, face, chest, upper extremities, lower extremities, and the spine. For the head, concussion with and without the loss of consciousness, skull fracture, and traumatic brain injury were all identified by the panel as critical injuries. For the face, eye and ear injuries, and facial fractures were all identified. Lung contusions, rib fractures, hemo-, pneumo-, and hemopneumothorax were classified as critical chest injuries. For upper and lower extremities, joint injury (including shoulder dislocation) and skeletal fracture were categorized as critical as well. Finally, for the spine (cervical, thoracic, and lumbar), brachial plexus injury, cord contusion, vertebral fracture, herniated disks, and disk rupture were all added to the list.

After working with the expert panel, and in the process of testing, the team determined that hip loads are of particular interest and concern for returning crewmembers, as spaceflight deconditioning can affect hip strength. Hip loads were added to the matrix (see Table 3) after the initial discussion with experts based on the risk of femoral head fracture due to lateral loads.

**Table 3:** **Matrix showing the injury metric chosen and the relationship with the critical injuries**.

	Head injury	Facial trauma	Cervical spine trauma	Blunt trauma	Lung contusion	Rib fracture	Hemopneumothorax	Upper extremity joint injury	Upper extremity fracture	Femoral head fracture	Thoracic spine trauma	Lumber spine trauma	Lower extremity joint injury	Lower extremity fracture
HIC 15	T/H													
BrIC	T/H													
Neck axial tension			T/H											
Neck axial compression			T/H											
Max chest deflection					T	T	T							
Lateral shoulder force (deflection)					T/W	T/W	T/W	T/W	T/W					
Acetabular lateral load										T/W				
Thoracic spine axial compression											T/H	T/H		
Ankle moments													T	
Contact limits/restrains (design constraint)		X		X				X	X				X	X

*H, Hybrid-III; T, THOR; W, WorldSID; X, design constraint*.

### Injury metric and anthropomorphic test device selection

Following the expert panel recommendations, a team was organized to determine the best ATD (and associated metrics) for validating against the critical injury list. The team (consisting of researchers from NHTSA, FAA, and NASA) began evaluating which ATD metrics were the best choices given the list of critical injuries and NASA’s dynamic environment. The team examined each anatomical region and identified available injury metrics that could address each critical injury. Each metric identified was associated with an ATD that provides that measurement capability (see Table 3).

Because IARVs are related to the particular ATD employed, the team considered all of the applicable ATDs available including the Hybrid-III, the WorldSID, and the test device for human occupant restraint (THOR). During discussions of the injury criteria, the team reviewed the benefits and drawbacks of each ATD as shown in Table 4. The final consensus was to use the 50th percentile THOR ATD as the primary ATD for testing and analysis. Although it is only currently available in one size and the current FEM is still preliminary, the team chose the THOR because of its biofidelity and multi-axis performance. To overcome the limitations of the THOR, IARVs will eventually be chosen that incorporate the increased risk of gender differences and various anthropometries. In addition, lateral tests comparing the 50th percentile WorldSID and the 50th percentile THOR are planned to determine if the THOR is a pragmatic choice for lateral testing. Although the THOR was not designed for side-impact conditions, the IRL has conducted side-impact testing using the THOR. If possible, the team would prefer to use the THOR in all axes to simplify analysis and testing; however, further evaluations of the THOR and WorldSID in lateral loading conditions will be necessary to validate this approach. In the event that the THOR is found to be inadequate in predicting injuries due to lateral loads, the team would recommend using the WorldSID for such cases.

**Table 4:** **Benefits and limitations of each ATD**.

	Hybrid-III		THOR		WorldSID	
	Benefit	Limitation	Benefit	Limitation	Benefit	Limitation
Injury criteria	Injury criteria readily available	Not representative of human responses	Improved neck biofidelity	Several injury criteria still in development	Improved shoulder biofidelity over EuroSID	Several injury criteria still in development
			Simulated neck muscle tension	
		Injury criteria not developed for low injury risk	Improved chest geometry and response		Improved chest biofidelity over EuroSID	
			Better frequency response	
			Better response at low dynamics	
FE model status	FE model commercially available	Not well validated in other loading directions		FE model is preliminary and does not reflect current design		No FE model available
Anthropometry	5th, 50th, 95th		5th^a^, 50th	Only one anthropometric size currently available	5th^a^, 50th	Only one anthropometric size available
Directionality	Validated in frontal impacts	Requires modification for *Z* axis^b^	Validated in frontal impacts	Unknown responses in *Y* and *Z* axes^c^	Validated in side-impacts	Not suited for *X* axis
		Not suited for *Y* axis	Closest ATD to multidirectional			
Availability	Readily available			Limited availability		Limited availability

*^a^Fifth percentile THOR and WorldSID currently in development. Will not be available for NASA use for several years*.

*^b^*Z* axis testing with the Hybrid-III requires modification (“Aerospace” model)*.

*^c^Used by the Indy racing league (IRL) in side-impact tests. Further testing required*.

### Injury assessment reference values as determined from a literature review

Having selected the ATD and injury metrics, work focused on the IARV associated with each metric. Since most available literature classifies injuries according to the AIS, the team decided to map the AIS level directly to each injury classification (Association for the Advancement of Automotive Medicine, 2005). The team used nominal and off-nominal risk levels from the definition of acceptable risk (DAR) to determine the appropriate IARVs for each metric as shown in Table 5 (Somers et al., 2014). In addition, for each IARV, a confidence score is included. This score is a qualitative estimate of the confidence in the IARVs reported and is on a scale of 0–5 (no confidence–full confidence, respectively).

**Table 5:** **Injury classification mapping and acceptable risk levels**.

Injury class	Acceptable risk level across all missions	
	Nominal (%)	Off-nominal (%)
Class I (AIS1+)	5	19
Class II (AIS2+)	1	4
Class III (AIS3+)	0.3	1
Class IV (AIS4+)	0.03	0.1

#### IARV for head injury criteria

The head injury criteria (HIC) are the standard head injury predictor in the automotive industry. Equation 1 is used to calculate the HIC (Prasad and Mertz, 1985).

Head injury criteria formula:

(1) $${\mathrm{HIC}}_{15}=\underset{0\leq t_{2}-t_{1}\leq 0.015}{\max}\;\left(\left(t_{2}-t_{1}\right){\left[{\int \;}_{t_{1}}^{t_{2}}a\left(t\right)dt\frac{1}{t_{2}-t_{1}}\right]}^{2.5}\right)$$

**Figure 2:**
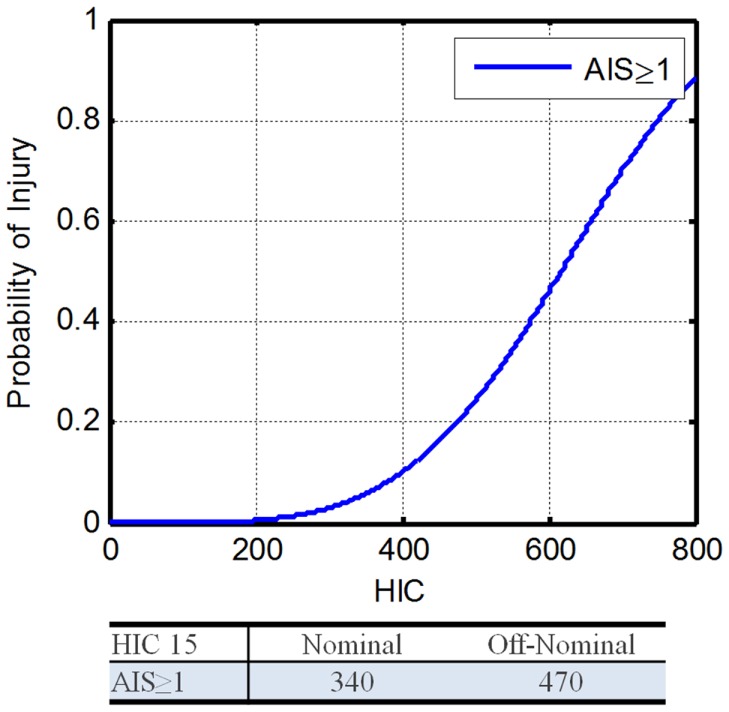
**Head injury criteria 15 injury risk function (Funk et al., 2007)**.

Recent studies of mild traumatic brain injury (mTBI) in football players can be very useful for determining the appropriate threshold for head injury. Since AIS 1 and 2 injuries to the brain are of primary concern, the HIC injury risk functions from Funk et al. (2007) will be used (Eq. 2). These data were chosen over the Virginia Tech data reported by Funk in 2012 because the HIC values from the 2007 study are more conservative (Funk et al., 2012). Because concussion injury risk determined from NASCAR head injury modeling resulted in much higher allowable HIC values (Somers et al., 2011), the Funk HIC 15 curve will be used to be conservative until the NASCAR results can be verified with additional datasets. In addition, the THOR ATD is assumed to have similar head kinematics as the Hybrid-III ATD. That allows for the use of identical HIC limits for both ATDs. Figure 2 shows the resultant HIC 15 injury risk function.

Head injury criteria injury risk model:

(2) $$p\left(\mathrm{inj}|\mathrm{AIS}\geq 1\right)=1-e^{-{\left(\frac{\mathrm{HIC}}{\beta }\right)}^{\alpha }}$$

Head injury criteria IARV model:

(3) $$\mathrm{IARV}\left(100\mathrm{p\%}|\mathrm{AIS}\geq 1\right)=\beta -\sqrt[{\alpha }]{\mathrm{In}\left(1-p\right)}$$

where, α is the cut point for the specified AIS level, β is the regression coefficient. Using Eq. 3 with α = 4.34 and β = 671, along with the DAR probabilities, the IARVs for nominal and off-nominal are 340 and 470, respectively (Table 6). Based on the available literature and its applicability to the environment expected during spaceflight, the confidence in these IARVs is rated at a 4 on a scale from 0 to 5, with 5 being the most confident.

**Table 6:** **Table of proposed THOR injury assessment reference values (IARV)**.

	Conditioned		Deconditioned		IARV confidence level (0–5)
	Nominal	Off-nominal	Nominal	Off-nominal	
HIC 15	340	470	340	470	4
BrIC	0.04	0.07	0.04	0.07	2
Neck axial tension force (N)	880	1,000	760^a^	860^a^	4
Neck axial compression force (N)	580	1,100	500^a^	950^a^	3
Max chest deflection (mm)	25	32	25	32	2
Lateral shoulder force (N)	2,700	3,300	2,700	3,300	4
Acetabular resultant force (N)	1,600	2,900	1,200^b^	2,200^b^	3
Thoracic spine axial compression force (N)	5,800	6,500	5,000^a^	5,600^a^	3
Ankle dorsiflexion moment (Nm)	18	31	14^b^	23^b^	3
Ankle inversion/eversion moment (Nm)	17	22	13^b^	17^b^	3
Average distal forearm speed (m/s)	8.1	10	8.1	10	3

*^a^Spinal deconditioning factor of 0.86 applied*.

*^b^Lower extremity deconditioning factor of 0.75 applied*.

#### IARV for kinematic rotational brain injury criteria

To further mitigate the risk of mTBI, an injury metric related to angular head dynamics was selected. Takhounts et al. (2013) reports the calculation method for deriving brain rotational injury criteria (BrIC). The revised BrIC can be applied regardless of the ATD used, so is applicable to the THOR. The BrIC is calculated using Eq. 4.

BrIC formula:

(4) $$\mathrm{BrIC =}\sqrt{{\left(\frac{\omega_{x}}{\omega_{xC}}\right)}^{2}+{\left(\frac{\omega_{y}}{\omega_{yC}}\right)}^{2}+{\left(\frac{\omega_{z}}{\omega_{zC}}\right)}^{2}}$$

where, ω*_i_* is the maximum angular head velocity in the *i* plane, ω*_iC_* is the angular head velocity critical value for the *i* plane.

**Figure 3:**
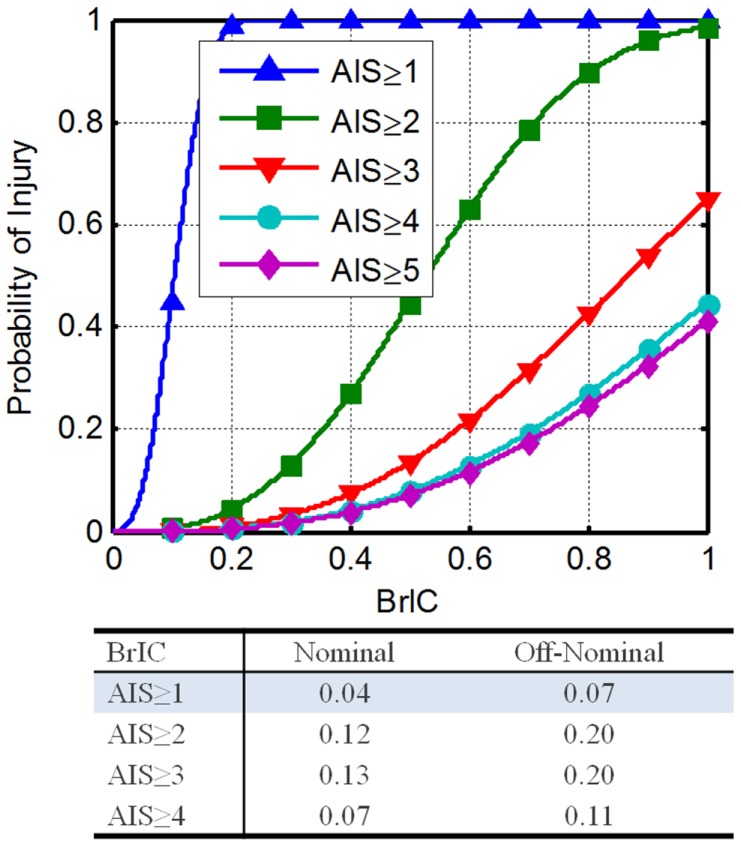
**Brain rotational injury criteria THOR injury risk functions (Saunders et al., 2012)**.

The critical values are 66.3, 53.8, and 41.5 rad/s for the *X, Y*, and *Z* axes. Equation 6 details how to determine IARVs for BrIC. Figure 3 shows the resultant injury risk curves.

BrIC injury risk model:

(5) $$p\left(\mathrm{AIS}\geq n|\mathrm{BrIC}\right)=1-e^{-{\left(\frac{\mathrm{BrIC}}{\lambda_{n}}\right)}^{2.84}}$$

BrIC IARV determination:

(6) $$\mathrm{IARV}\left(100\mathrm{p\%}|\mathrm{AIS}\geq n\right)=\lambda_{n}\cdot \sqrt[{2.84}]{-\log\left(1-p\right)}$$

where, *n* is the specified AIS level, λ*_n_* is the scale parameter for the specified AIS level.

Using Eq. 6 with the associated scale and shape parameter values, along with the DAR probabilities, the IARVs in Table 6 can be derived. To satisfy the desired probabilities of injury associated with all four AIS levels, the minimum BrIC for nominal and off-nominal conditions was selected. For nominal and off-nominal conditions, a BrIC of 0.04 and 0.07, respectively, are required so that all four AIS levels are satisfied. Although these values are most conservative, the BrIC was developed for AIS ≥ 4 injury levels and then scaled to other AIS levels. Based on the uncertainty associated with the extrapolation of the BrIC for low injury risk, the confidence in these IARVs is rated at a 2.

#### IARV for neck axial tension

The THOR neck is designed to mimic the human neck response by meeting biofidelity goals based on performance corridors from Mertz et al. (1997), Naval Biodynamics Laboratory (NBDL), and Japan Automotive Research Institute (JARI) human volunteer tests (White et al., 1996). Although the goal was to produce a neck that is biofidelic in all loading conditions, the axial stiffness is greater than desired (Dibb et al., 2006). However, the axial performance of the neck in loading through the center of gravity (CG) can be related to the computation model of PMHS. This is expected to be the primary loading expected in NASA landings due to inertial loads on the head. The computational model used was a validated finite element (FE) model of the human neck. Note that the THOR Mod Kit ATD was revised to behave similar to the THOR-NT without muscle cables case, which correlates well with the computational model. Using this relationship, a transfer function between the THOR Mod Kit and the computational model can be determined (Eq. 9).

THOR axial tension relationship to applied force:

(7) $$F_{z\mathrm{THOR}}=0.8228\cdot F_{\mathrm{applied}}$$

PMHS axial tension relationship to applied force:

(8) $$F_{z\mathrm{PMHS}}=0.5983\cdot F_{\mathrm{applied}}$$

THOR axial tension relationship to PMHS:

(9) $$F_{z\mathrm{THOR}}=1.38\cdot F_{\mathrm{PMHS}}$$

**Figure 4:**
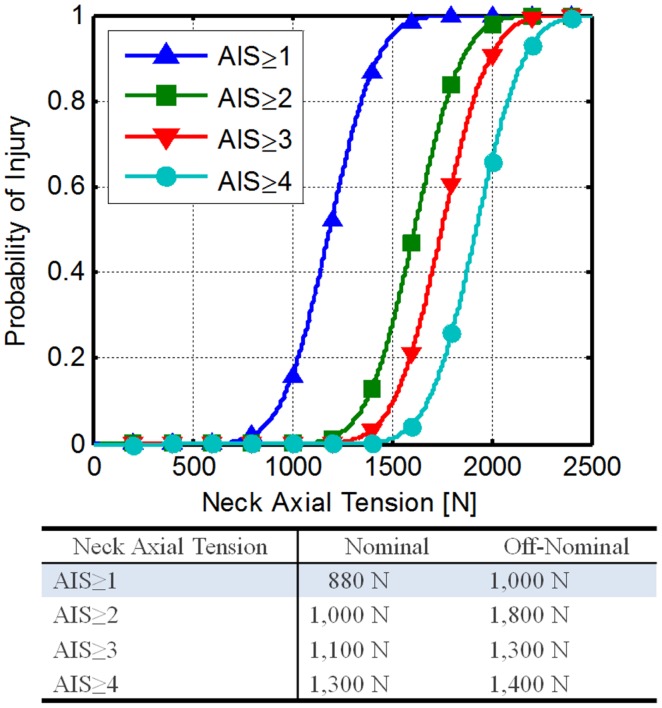
**Neck axial tension force risk functions developed fromPhilippens et al. (2011)**.

A study reported by Philippens et al. (2011) was performed to determine the axial tension force injury risk function associated with PMHS injuries. Using an ordered probit analysis on the maximum reported AIS (anatomical and clinical), the risk functions shown in Eqs 10 and 11, and Figure 4 were developed.

Neck tension injury risk:

(10) $$p\left(\mathrm{AIS}\geq n|F_{z}\right)=1-\Phi \left(c_{n}-F_{z}\cdot \beta \right)$$

Neck tension injury risk model:

(11) $$\mathrm{IARV}\left(\mathrm{AIS}\geq n|p\right)=\frac{c_{n}-\Phi^{-1}\left(1-p\right)}{\beta }$$

where, *n* is the specified AIS level, Φ is the standard Gaussian distribution, *C_n_* is the cut point for the specified AIS level, *c*_1_ = 6.30, *c*_2_ = 8.56, *c*_3_ = 9.28, *c*_4_ = 10.19, and β is the regression coefficient (0.0053).

Using Eqs 9 and 11 along with the DAR probabilities, the IARVs in Table 6 can be derived. The AIS ≥ 1 IARVs (880 and 1,000 N for nominal and off-nominal conditions, respectively) are lowest, and are selected to be conservative.

National Highway Traffic Safety Administration is proposing an IARV of 2,520 N for neck axial tension for automotive use and relates to a 22% risk of an AIS ≥ 3 injury (Dibb et al., 2006). Based on the assumptions made and the comparison to the NHTSA IARVs, the confidence in these IARVs is rated at a 4.

#### IARV for neck axial compression

Neck compression is of particular concern during spacecraft landing, because there can be a significant +*Z* acceleration causing neck compression from the inertial effects of the head. In addition, any head-mounted mass, such as a conformal helmet, could increase the load on the neck.

As with neck tension, the assumption was made that the THOR neck is biofidelic, and thus cadaveric injury risk functions can be directly applied. Although this may not be the case, it is assumed that the mechanical neck of the THOR would be stiffer than the human (similar to the trend seen in neck tension). Pintar et al. (1998a) conducted an experiment consisting of nine male and four female cervical PMHS spines to developed neck compression injury risk functions for axial compression. The mean age of the subjects was 59 years old (range of 39–82). These data were collected during hyperflexion, which is the cause of 48–70% of neck injuries (Allen et al., 1982; Yoganadan et al., 1989). As this represents the most likely mechanism for injury and is the most conservative for neck compression, these data will be used to develop neck compression IARVs.

**Figure 5:**
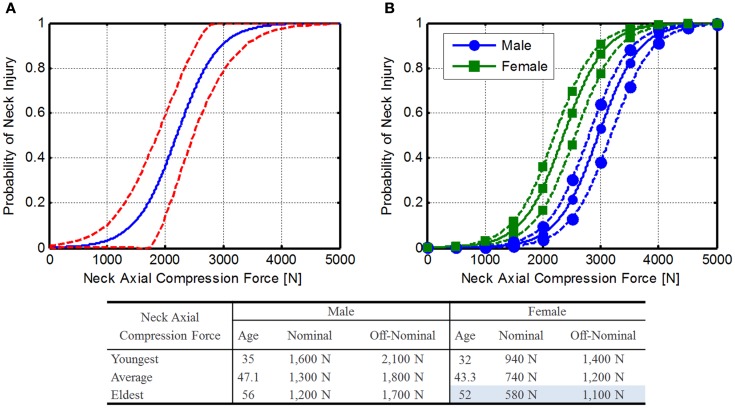
**Neck axial compression force injury risk function**. **(A)** Original Pintar risk function (Pintar et al., 1998a) with confidence limits (dashed lines) and **(B)** expanded risk functions for male and female astronauts. Solid line represents mean astronaut age (47.1 and 43.3 for males and females, respectively), dashed lines represent the youngest and oldest astronauts (male: 35 and 56; female: 32 and 54). Note that force tolerance declines with age.

Pintar et al. (1998b) report an injury risk function based on cervical compression force (Figure 5A). Using this risk function as a starting point, the results of another Pintar et al. (1990a,b, 1998a) study were investigated, which was based on data collected previously. Equation 12 gives the 50% injury risk function based on multiple factors including loading rate, age, and gender. Combining the logistic regression equation with Eq. 12 gives Eq. 13. This equation can then be rewritten to determine injury risk based on a neck axial compression force (Eq. 14).

Neck compression fracture tolerance model:

(12) $$F_{z}=\beta_{0}+\beta_{1}\cdot A+\beta_{2}\cdot \mathrm{LR+}\beta_{3}\cdot G+\beta_{4}\cdot A\cdot \mathrm{LR}$$

where, β_0_ is the constant coefficient with a value of 934.2, β_1_ is the age coefficient with a value of 8.9, β_2_ is the loading rate coefficient with a value of 11.0, β_3_ is the gender coefficient with a value of 665.0, and β_4_ is the age and loading rate coefficient with a value of −0.134.

Neck compression injury risk:

(13) $$p\left(\mathrm{AIS}\geq 2|F_{z}\right)=\frac{1}{1+e^{\beta_{5}\left(\beta_{0}+\beta_{1}\cdot A+\beta_{2}\cdot \mathrm{LR+}\beta_{3}\cdot G+\beta_{4}\cdot A\cdot \mathrm{LR}-F_{z}\right)}}$$

Neck compression injury risk model:

(14) $$\begin{array}{llll} \mathrm{IARV}\left(\mathrm{AIS}\geq 2|p\right) & =\beta_{0}+\beta_{1}\cdot A+\beta_{2}\cdot \mathrm{LR}\; & & \;\\ & \;+\beta_{3}\cdot G+\beta_{4}\cdot A\cdot \mathrm{LR+}\frac{\log\left(\frac{1}{p}-1\right)}{\beta_{5}} \end{array}$$

where, *F_Z_* is the peak neck axial compression (N), *p* is the probability of neck injury, β*_n_* are the model coefficients, *A* is the subject age (years), LR is the loading rate (m/s), *G* is 0 for females, and 1 for males.

The average age of the current astronaut population is 47.1 years for males (range 35–56) and 43.3 years for females (range 32–52). To determine injury risk, 2 m/s was selected as the loading rate. This rate was selected to give conservative numbers, since young females are more likely to suffer injury due to the lower loading rate. Using these ages along with a loading rate of 2 m/s, an injury risk function can be determined (Figure 5B), and the IARVs in Table 6 can be derived using the DAR probabilities. In this case, older females have the lowest tolerance to neck compressive force, so these values will be used as the IARVs (580 and 1,100 N for nominal and off-nominal, respectively).

National Highway Traffic Safety Administration is proposing an IARV of 3,640 N for neck axial compression for automotive use and relates to a 22% risk of an AIS ≥ 3 injury (Dibb et al., 2006). Based on the assumptions made and the comparison to the NHTSA IARVs, the confidence in these IARVs is rated at a 3.

#### IARV for chest deflection

**Figure 6:**
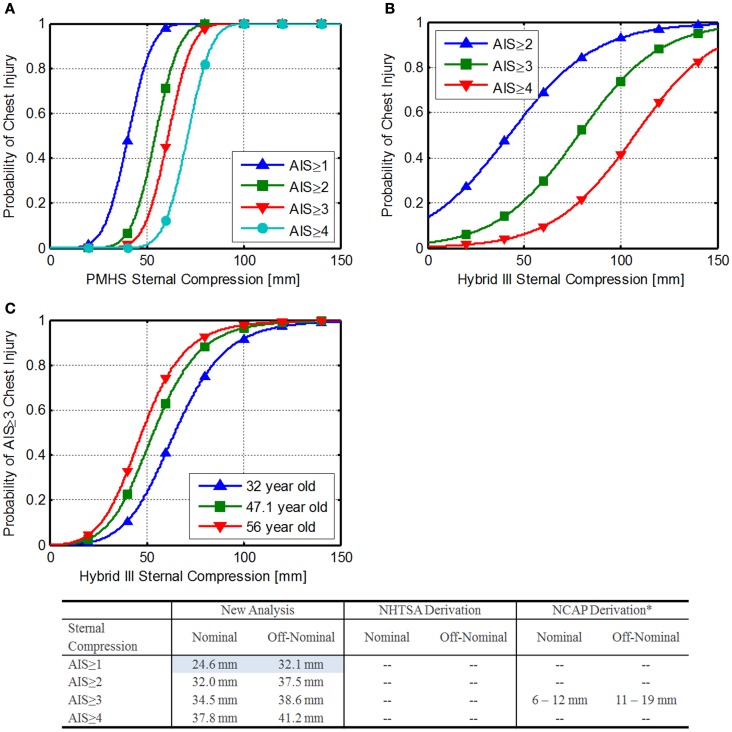
**Sternal compression injury risk**. **(A)** Injury risk functions developed from Mertz et al. (1997), **(B)** NHTSA FVMSS 208 injury risk functions (National Highway Traffic Safety Administration, 1998), and **(C)** NHTSA NCAP chest injury risk by age (National Highway Traffic Safety Administration, 2008).

To develop sternal compression IARVs, the assumption was made that the THOR thorax is biofidelic (Neathery, 1974; General Engineering and Systems Analysis Company, 2005). Mertz et al. (1997) reported sternal compression and injury data from PMHSs. Based on the methods detailed in Somers et al. (2011) an ordered probit analysis was used on the reported AIS, resulting in the risk functions shown in Eq. 15 and Figure 6A.

Sternal compression injury risk:

(15) $$p\left(\mathrm{AIS}\geq n|D_{\mathrm{chest}}\right)=1-\Phi \left(c_{n}-D_{\mathrm{chest}}\cdot \beta \right)$$

Sternal compression IARV calculation:

(16) $$\mathrm{IARV}\left(\mathrm{AIS}\geq n|p\right)=\frac{c_{n}-\Phi^{-1}\left(1-p\right)}{\beta }$$

where, *n* is the specified AIS level, Φ is the standard Gaussian distribution, *c_n_* is the cut point for the specified AIS level, *c*_1_ = 4.17, *c*_2_ = 5.61, *c*_3_ = 6.29, *c*_4_ = 7.32, and β is the regression coefficient (0.103).

Using Eq. 16 along with the DAR probabilities, the IARVs in Table 6 can be derived. The AIS ≥ 1 IARVs are lower, and are selected to be conservative.

National Highway Traffic Safety Administration uses the formulas in Eqs 17–20 to estimate injury risk related to sternal compression on the Hybrid-III (National Highway Traffic Safety Administration, 1998). Based on Eq. 18, a sternal compression of 63 mm corresponds to a 33% risk of an AIS ≥ 3 injury. Figure 6B shows the injury risk functions related to sternal compression. IARVs are derived in the table in Figure 6. Note that the lower injury risk curves cross zero above the desired injury risk levels, so they are not reported.

Risk of AIS ≥ 2 chest injury:

(17) $$p\left(\mathrm{AIS}\geq 2|D_{\max}\right)=\frac{1}{1+e^{1.8706-0.04439D_{\max}}}$$

Risk of AIS ≥ 3 chest injury:

(18) $$p\left(\mathrm{AIS}\geq 3|D_{\max}\right)=\frac{1}{1+e^{3.7124-0.0475D_{\max}}}$$

Risk of AIS ≥ 4 chest injury:

(19) $$p\left(\mathrm{AIS}\geq 4|D_{\max}\right)=\frac{1}{1+e^{5.0952-0.0475D_{\max}}}$$

Risk of AIS ≥ 5 chest injury:

(20) $$p\left(\mathrm{AIS}\geq 5|D_{\max}\right)=\frac{1}{1+e^{8.8274-0.0459D_{\max}}}$$

The NHTSA new car assessment program (NCAP) has an additional injury risk function for AIS ≥ 3 injury risk (National Highway Traffic Safety Administration, 1998) (Eq. 21; Figure 6C).

NHTSA NCAP AIS ≥ 3 injury risk:

(21) $$p\left(\mathrm{AIS}\geq 3|D_{\max},\mathrm{Age}\right)=\frac{1}{1+e^{12.597-0.05861\cdot \mathrm{Age}-1.568{\left(D_{\max}\right)}^{0.4612}}}$$

Yaguchi et al. (2009) report similar chest deflections between the Hybrid-III and the THOR-NT, making these numbers applicable to the THOR, although in comparison to the values calculated previously, these appear to be highly conservative. The conservative results may be attributable to the lack of fidelity at the very low risk of injury.

Based on the results of the three analyses, the chest compression IARVs from the Mertz re-analysis are chosen for inclusion in Table 6 and are 24.6 and 32.1 mm for nominal and off-nominal, respectively.

Based on the assumptions made, the limited data used to develop these limits, and the discrepancy between the NHTSA values, the confidence in these IARVs is rated at a 2.

#### IARV for lateral shoulder force and displacement

The THOR ATD was not intended for the use as side-impact dummy (SID); however, there is evidence that it can be used in side-impact testing (Rangarajan et al., 2000). There is very little information on injury related to lateral shoulder force. Two methods are described for estimating an IARV for this metric.

##### Method 1 comparison to Brinkley *Y* dynamic response

**Figure 7:**
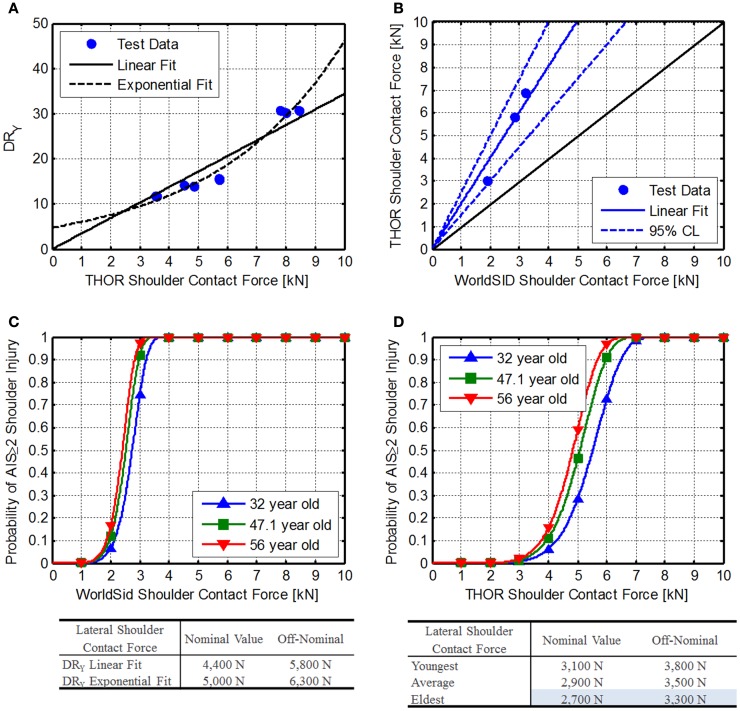
**Shoulder contact force**. **(A)** Comparison of THOR shoulder contact force related to the BDRC DR_y_, **(B)** Comparison of WorldSID and THOR shoulder contact force data reported by Pintar et al. (2007), **(C)** WorldSID injury risk function reported by Petitjean et al. (2012), and **(D)** developed THOR shoulder force injury risk functions.

Using data collected with the THOR during testing at Wright–Patterson Air Force Base, the THOR shoulder contact force can be correlated with the *Y* axis Dynamic Response. Two different fits were used to determine if the fit had an appreciable effect on the limits (Figure 7A). The linear fit was constrained to pass through zero.

THOR shoulder contact force linear fit to DR_y_:

(22) $$DR_{y}=3.43\cdot 10^{-3}\cdot F_{y}$$

THOR shoulder contact force exponential fit to DR_y_:

(23) $$DR_{y}=4.815\cdot e^{0.000226\cdot F_{y}}$$

##### Method 2: comparison to WorldSID side-impact dummy

Pintar et al. (2007) reported identical tests conducted with the THOR-NT ATD and the WorldSID. Using data from configuration 4, 5, and 16, shoulder contact force can be compared between the THOR-NT and the WorldSID for 90° impact conditions (Figure 7B) using Eq. 24.

THOR–WorldSID shoulder contact force transfer function:

(24) $$F_{\mathrm{THOR}\;\mathrm{Contact}}=2.016\cdot F_{\mathrm{WorldSID}\;\mathrm{Contact}}$$

Petitjean et al. (Figure 7C) reported WorldSID shoulder injury risk function related to should contact force and age (Petitjean et al., 2012).

WorldSID shoulder contact force injury risk:

(25) $$\mathrm{Risk}\left(\%\right)=1-e{\left[-\frac{F}{e^{8.14-0.0055\cdot \mathrm{age}}}\right]}^{7.41}$$

WorldSID shoulder contact force IARV calculation:

(26) $$F=e^{8.14-0.0055\cdot \mathrm{age}}\cdot \sqrt[{7.41}]{-\ln\left(1-\mathrm{Risk}\right)}$$

Combining Eqs 24 and 26, Eq. 27 can be derived.

THOR shoulder contact force IARV calculation:

(27) $$F=2.016\cdot e^{8.14-0.0055\cdot \mathrm{age}}\cdot \sqrt[{7.41}]{-\ln\left(1-\mathrm{Risk}\right)}$$

Using current astronaut ages (minimum, maximum, and average), AIS ≥ 2 IARVs can be determined from Eq. 27 (Figure 7D). Based on the values, the eldest astronaut IARVs are the lowest and chosen to be most conservative (Table 6) and are 2,700 and 3,300 N for nominal and off-nominal, respectively. Based on the available information in the literature, the confidence in these IARVs is rated at a 4.

#### IARV for lateral acetabular force

For lateral loads, the pelvic restraint design may not contact the iliac crest, concentrating the load on the femoral head and neck, which is at a greater risk of fracture after spaceflight (Lang et al., 2004). Because of this concern, a desirable feature of the THOR ATD is the acetabular load cells built into the pelvis structure. Four methods are compared to estimate the IARV for this measure.

##### Method 1: pelvis lateral force comparison

A comparison of applied lateral pelvic force is made between the THOR and EuroSID ATDs, allowing injury risk to be extrapolated to the THOR lateral force. Although this does not directly translate to acetabular forces, if the iliac crest is not engaged, it should be a comparable load into the acetabulum.

**Figure 8:**
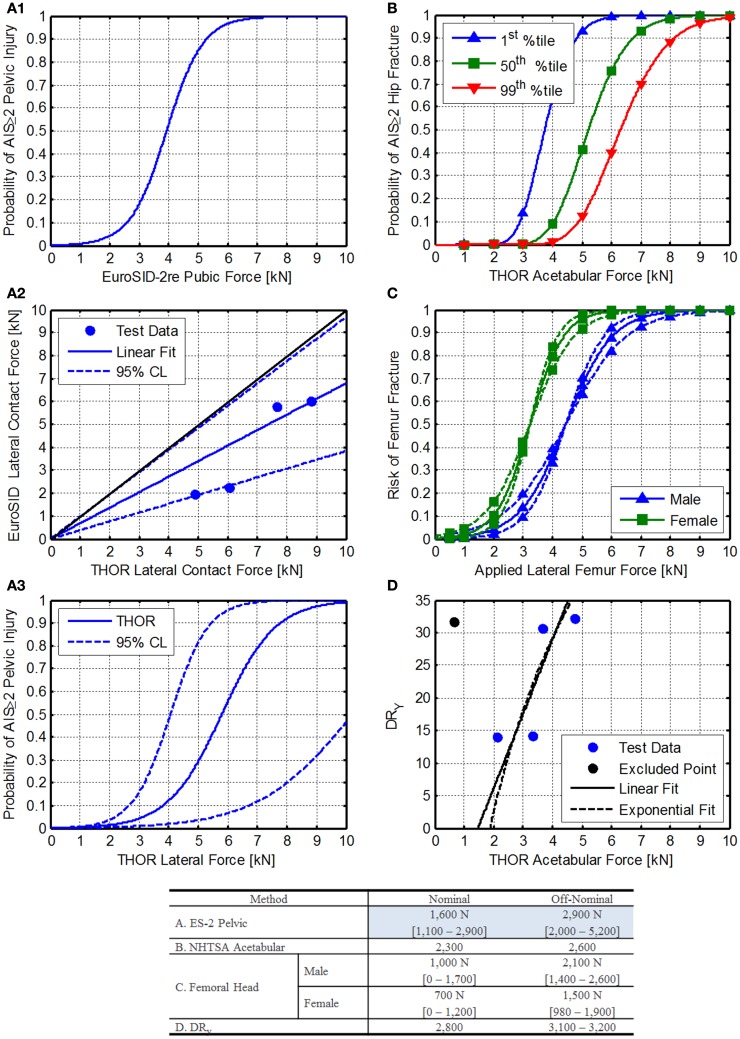
**Lateral acetabular force injury risk function development**. **(A1)** EuroSID 2-re pelvic injury risk function reported by Kuppa (2004). **(A2)** Comparison of EuroSID and THOR lateral contact force. Blue dots represent matched pairs of test data, black line represents an equal response from both ATDs, and the blue line (with dotted lines) represent the best linear fit of the data through zero with 95% confidence limits shown as dashed lines. **(A3)** THOR injury risk function for lateral contact force, **(B)** NHTSA acetabular injury risk function for frontal impact as reported by Rupp et al. (2010). **(C)** Femur fracture risk associated with applied forces in humans. The solid lines are the injury risk estimate, and the dashed lines are the 95% confidence interval. **(D)** Comparison of the THOR acetabular loads and the BDRC DR_Y_. The blue dots are test data, the black line is the linear fit, and the dashed black line is an exponential fit.

Kuppa (2004) shows the risk of injury related pubic force in the EuroSID-2re ATD (Eq. 28; Figure 8A1).

Pelvic injury risk function for the EuroSID-2re (Kuppa, 2004):

(28) $$p\left(\mathrm{AIS}\geq 2|F_{\mathrm{EuroISD}}\right)=\frac{1}{1+e^{\left(6.403-0.00163\cdot F_{\mathrm{EuroISD}}\right)}}$$

Because there are no data currently published in the literature to relate the THOR directly to PMHS lateral applied forces, a comparison of THOR to EuroSID is used instead.

Data collected from testing conducted at the Air Force Research Laboratory on the horizontal impulse accelerator (HIA) is used to compare the lateral forces imparted on the lateral side support during lateral tests. The tests were paired based on the peak acceleration for each test. For the 10G tests, only 1 test was completed with the THOR, so it was used for comparison to both EuroSID tests. The comparison is shown in Figures 8A2,A3 and the transfer function is described in Eq. 29.

Transfer function between THOR-K and EuroSID-2re lateral contact force:

(29) $$F_{\mathrm{EuroSID}}=1.47\cdot F_{\mathrm{THOR}}$$

Using the transfer function between the THOR and EuroSID the injury risk function can be determined from Eq. 28, resulting in Eq. 30. The injury risk function is shown in Figure 8A3 along with the 95% confidence limits. The IARVs are shown in the table in Figure 8. Note that these values are for risk of injury to the pelvis due to pelvic loading and may underestimate the risk to the acetabulum and femoral head and neck.

Risk of injury due to lateral force on the THOR ATD:

(30) $$p\left(\mathrm{AIS}2+\right)=\frac{1}{1+e^{\left(6.4.3-0.0011\cdot F_{\mathrm{THOR}}\right)}}$$

##### Method 2: THOR frontal impact acetabular injury risk

National Highway Traffic Safety Administration is developing on an acetabular injury risk function based on research performed by Rupp et al. (2010). These forces are intended for axial loading of the femur, which is a different load path than the expected lateral loading of the acetabulum as expected with spacecraft. The assumption is made that lateral loading into the acetabulum has a similar risk of injury, although there are no experimental data to substantiate this assumption. The formula provided by NHTSA is given in Eq. 31.

Acetabular injury risk:

(31) $${\mathrm{Risk}}_{\mathrm{Hip}\;F_{x}}=\Phi \left[\frac{\ln\left[F_{\mathrm{res}}\right]-\ln\left[e^{-0.2141+0.0114\cdot s}\cdot \left(1-\frac{\left(f-a\right)}{100}\right)\right]}{0.1991}\right]$$

where, Φ is the cumulative distribution function of the standard normal distribution, *F* is the peak force transmitted to the hip (in kN), *S* is the stature of the target population for the risk curve (for 50th percentile males, 178 cm), *f* is the hip flexion angle (in degrees; neutral posture = 30°), and *a* is the hip abduction angle (in degrees; neutral posture = 15°).

Figure 8B shows the injury risk functions for 1st, 50th, and 99th percentile crewmembers. Since the first percentile female values are lowest, they were chosen for inclusion to be conservative.

##### Method 3: comparison to human femoral head injury risk

Nelson et al. (2009) describe a method for estimating bone injury risk based on the fracture risk index (FRI) as shown in Eq. 32. FRI can be described by Eq. 33, giving Eq. 34. Although this may predict the risk of injury in a human, there is little known about the correlation of these forces to the ATD acetabulum. Because the THOR femur is not as compliant as bone, different forces at the acetabulum would be expected in the same loading conditions.

Probability of fracture risk:

(32) $$p_{\mathrm{fracture}}=\frac{1}{1+e^{-\left(\mathrm{FRI}-\mu \right)\cdot \phi }}$$

Fracture risk index:

(33) $$\mathrm{FRI}=\frac{F_{\mathrm{Applied}}}{F_{\mathrm{fracture}}}$$

Probability of fracture risk:

(34) $$p_{\mathrm{fracture}}=\frac{1}{1+e^{-\left(\frac{F_{\mathrm{Applied}}}{F_{\mathrm{fracture}}}-\mu \right)\cdot \phi }}$$

where, *F*_Applied_ is the force applied to the femur, ϕ is the slope factor (a measure of the steepness of the curve), and μ is the position factor of the curve (the value of FRI where the probability is 0.50).

Nelson et al. (2009) give estimates of μ and ϕ, which were derived from pediatric radial arm fractures. In addition, Cheng et al. (1997) give fracture forces for males and females. Because the mean age is 67 and 71 for males and females, respectively, the lower 95th percentile values are used. The applied forces were then related to the injury risk levels for AIS ≥ 2 injuries (Figure 8C).

##### Method 4: comparison to DR_y_ values

The BDRC is currently used by NASA to protect crewmembers from dynamic loads. The BDRC was developed by the U.S. Air Force and is an evolution of previous attempts to quantify injury risk due to accelerations on the body (Brinkley et al., 1990). Although the *Y* axis model is based on limited data, it is useful to compare the DR_Y_ to data collected on the THOR at Air Force Research Laboratory recently. One case was excluded from the analysis, as the run included significant pelvis involvement (high pelvis acceleration), indicating the load path was not through the acetabular joint. Figure 8C shows the relationship between the DR_Y_ and acetabular force along with the associated fits (Eqs 35 and 36). Based on these results and the current DR_Y_ limits, IARVs can be developed.

Linear THOR acetabular fit:

(35) $$F_{y}=0.0876\cdot DR_{y}+1.464$$

Exponential THOR acetabular fit:

(36) $$F_{y}=1.867\cdot e^{0.02595\cdot DR_{y}}$$

##### Summary

Figure 8 shows the comparison of the four methods used to develop IARVs for the acetabular force. Given the wide variability, particularly of the femoral head IARVs, the ES-2 pelvic method is chosen (Table 6) since it is more conservative than all of the other methods except the femoral head method. The IARVs are 1,600 and 2,900 N for nominal and off-nominal, respectively. Based on the range of values determined in each of the four analyses and the level of extrapolation, the confidence in these IARVs is rated at a 3.

#### IARV for thoracic spine axial force

**Figure 9:**
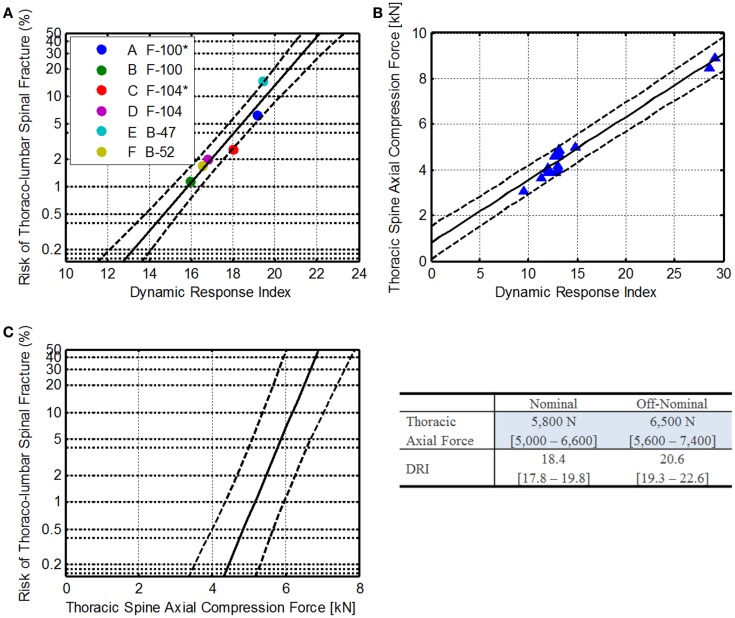
**Thoracic spine axial compression injury risk development**. **(A)** Relationship of DRI to operational injury data, where * denotes rocket assisted ejections (Brinkley, 1968; Brinkley and Schaffer, 1971). **(B)** Comparison of THOR thoracic spine axial compression to the DRI. The blue dots are individual sled tests, the solid line is the linear fit, and the dashed lines are the 95% confidence. **(C)** The resulting injury risk function for THOR thoracic spine axial compression force.

Currently, the BDRC is used to assess astronaut injury risk. The +*Z* axis model in the BDRC is also known as the dynamic response index (DRI). The DRI has been validated for predicting thoraco-lumbar spinal injury in military aircraft ejections (Figure 9A) (Brinkley, 1968; Brinkley and Schaffer, 1971). Using a base-10 log transformation, a linear fit was applied to these data to determine the relationship between DRI and injury.

Equation 37 shows the relationship between DRI and spinal injury risk. Solving for DRI, Eq. 38 can be derived.

Spinal injury risk based on DRI:

(37) $$p\left(\mathrm{AIS}\geq 1\right)=10^{\left(\frac{\mathrm{DRI}-15.8}{3.73}\right)}$$

DRI relationship to spinal injury risk:

(38) $$\mathrm{DRI}\left(p\right)=3.73\cdot \log_{10}\left(p\right)+15.8$$

Because the DRI does not specify injury severity, the equation is used to predict the risk of any injury.

Recently, vertical tests of the THOR ATD were conducted at the Air Force Research Laboratory on the Horizontal Impact Accelerator. Thoracic spine axial compression force and pelvis accelerations (along with other data channels) were collected. Using the pelvis accelerations, the DRI for each drop case was calculated. The results are shown in Figure 9.

Using a linear regression, the relationship between the axial force and DRI was identified (Eq. 39). Solving for DRI, Eq. 40 can be derived.

THOR thoracic axial force relationship with DRI:

(39) $$F_{Z}\left(\mathrm{DRI}\right)=0.277\cdot DRI+0.795\left[kN\right]$$

DRI relationship to spinal axial force:

(40) $$\mathrm{DRI}\left(F_{z}\right)=3.62\cdot F_{z}-2.59$$

Combining Eqs 38 and 40, the relationship between the thoracic axial compression force and spinal injury risk can be developed (Eqs 41 and 42). The estimated injury risk function is shown in Figure 9C.

Injury risk relationship to thoracic axial compression:

(41) $$p\left(\mathrm{spinal}\;\mathrm{injury}|F_{z}\right)=10^{\left(\frac{F_{z}-5.08}{0.03}\right)}$$

Thoracic axial compression force injury risk:

(42) $$F_{z}\left(\mathrm{spinal}\;\mathrm{injury}|p\right)=1.03\cdot \log_{10}\left(p\right)+4.93$$

Finally, using Eq. 42, thoracic axial compression force limits can be estimated (Table 6). The resulting IARVs are 5,800 and 6,500 N for nominal and off-nominal, respectively.

Based on the assumptions made and the available data, the limits described are within the range of operational data presented in the literature; however, because the THOR data are limited to only a few DRI levels, the confidence in these IARVs is rated at a 3. Additional THOR testing in the DRI range of 15–25 would provide more insight into the linearity of the THOR response.

#### IARV for ankle moments

Kuppa et al. (2001) developed injury risk functions for ankle moments. Below is a description of the IARVs for NASA’s use.

##### Dorsiflexion

**Figure 10:**
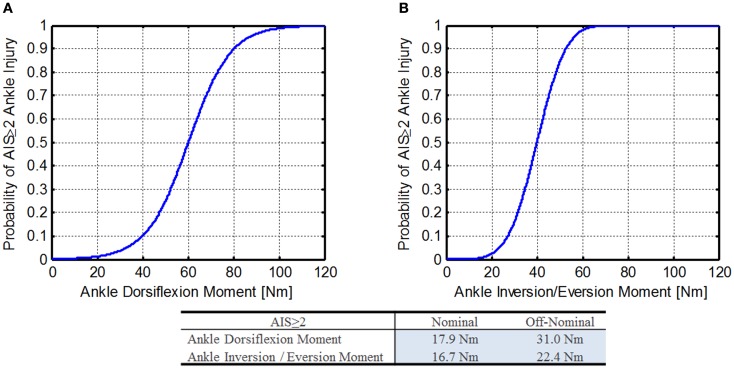
**Ankle moment injury risk functions (Kuppa et al., 2001)**. **(A)** Dorsiflexion ankle moment and **(B)** inversion/eversion ankle moment.

The dorsiflexion injury risk model is shown in Eq. 43 and Figure 10A.

Ankle dorsiflexion moment injury risk:

(43) $$p\left(\mathrm{AIS}\geq 2|M\right)=\frac{1}{1+e^{-\left(\frac{M-c}{\beta }\right)}}$$

Ankle dorsiflexion moment injury risk formula:

(44) $$\mathrm{IARV}\left(\mathrm{AIS}\geq 2|p\right)=c-\beta \cdot \log\left(\frac{1}{p}-1\right)$$

where, *p* is the specified injury probability, *M* is the applied dorsiflexion moment, *c* is the cut point (60.23), and β is the regression coefficient (9.217).

Using Eq. 44 along with the DAR probabilities, the IARVs in Table 6 can be derived and are 18 and 31 Nm for nominal and off-nominal, respectively.

##### Inversion/eversion

Kuppa et al. did not report the injury risk function for inversion/eversion ankle bending moment, but instead included a figure. Using a digitized and smoothed curve image from paper (Figure 10B), a probit function was fit to the curve (Eq. 45).

Ankle inversion/eversion moment injury risk:

(45) $$p\left(\mathrm{AIS}\geq 2\;\mathrm{Ankle}\;\mathrm{Injury}|M\right)=1-\Phi \left(c-\beta \cdot M\right)$$

Ankle inversion/eversion moment injury risk formula:

(46) $$\mathrm{IARV}\left(\mathrm{AIS}\geq 2|p\right)=\frac{c-\Phi^{-1}\left(1-p\right)}{\beta }$$

where, *M* is the ankle inversion/eversion moment, *p* is the specified injury probability, *c* is the cut point (4.0), and β is the regression coefficient (0.10).

Using Eq. 46 along with the DAR probabilities, the IARVs in Table 6 can be derived and are 17 and 22 Nm for nominal and off-nominal, respectively.

Based on the assumptions made and the extrapolation of the injury risk functions at lower injury risk levels, the confidence in these IARVs is rated at a 3.

#### IARV for upper extremity flail

During dynamic flight phases there is potential for extremity flail injury, which includes crewmember extremities impacting vehicular surfaces or objects, hyper-extending, hyper-flexing, hyper-rotating, fracturing, or dislocating without proper design consideration. For spacecraft operations, lower extremity range of motion isn’t necessary, so appropriate restraints will be required. For upper extremities, however, crewmembers need to be able to reach controls, and so upper extremity restraints must allow some limited range of motion, but also prevent contact with vehicular surfaces and restrain the limbs to remain within the seat envelop to prevent hyperextension, hyperflexion, and hyperrotation injuries.

Three approaches are discussed below for mitigating injury risk to upper extremity flail: (1) extremity contact moment limits, (2) average distal forearm speed (ADFS), and (3) active bracing limit. Each will be discussed below, and one method will be down-selected for inclusion in Table 6.

##### Method 1: extremity moment contact limits

**Figure 11:**
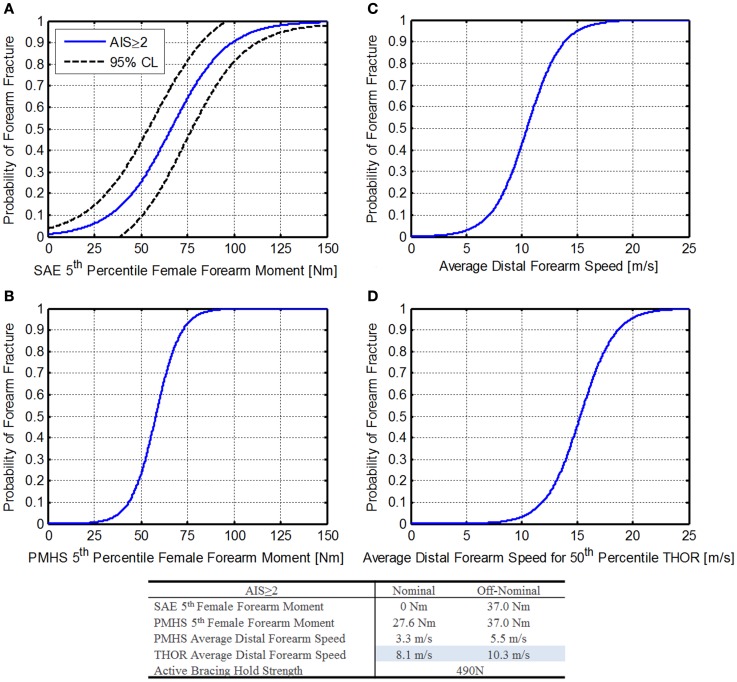
**Forearm risk function**. **(A)** SAE fifth percentile female forearm moment (Bass et al., 1997), **(B)** PMHS fifth percentile female forearm moment (Duma et al., 1999, 2003), **(C)** PMHS average distal forearm speed (Hardy et al., 1997), and **(D)** THOR average distal forearm speed.

In the late 1990s, before the change to de-powered airbags, automotive researcher found an increase in forearm injuries related to airbag deployment. To investigate forearm interaction with airbags, Bass et al. (1997) conducted PMHS studies relating forearm fractures with moments measured in the society of automotive engineers (SAE) fifth percentile female instrumented arm. The risk of a single forearm fracture is given by Eq. 47 and shown in Figure 11A. Note that this injury risk function is only applicable to the SAE instrumented arm.

Forearm moment injury risk:

(47) $$p\left(\mathrm{AIS}\geq 2|M\right)=\frac{1}{1+e^{-\left(\frac{M-66.2}{15}\right)}}$$

where, *M* is the Moment applied to the forearm.

Using the DAR probabilities of 1% for nominal and 4% for off-nominal (for AIS ≥ 2 injuries), forearm moments are 0 and 18.6 Nm, respectively. This is due to the use of a logistic regression that does not pass through zero. Unfortunately, because the THOR is only available in 50th percentile male size, this instrumented arm is not compatible.

Duma et al. (1999, 2003) report upper extremity moments related to injury risk for a fifth percentile female PMHS. Based on this injury risk, contact forces can be derived. These values will be conservative, as they assume that the contact force occurs at a point that induces the greatest moment, and in reality, contact forces closer to the joint may be higher and still induce a lower moment. Duma et al. reports upper extremity injury risk for other areas of the upper extremities; however, the forearm was selected as it represents the limiting case for contact force. Based on the results from Duma et al., Eq. 48 may be used to determine the injury risk associated with a forearm moment (Figure 11B).

Forearm injury risk:

(48) $$p\left(\mathrm{AIS}\geq 2|M\right)=\frac{1}{1+e^{-\left(\frac{M-58}{6.62}\right)}}$$

where, *M* is the moment applied to the forearm.

Using Eq. 48 along with the DAR probabilities, forearm moments are 27.6 and 37.0 Nm, respectively. Of note, these values are only valid for PMHS testing (Duma et al., 1999, 2003) and a further discussion can be found in the third edition of “Trauma biomechanics: accidental injury in traffic and sports” (Schmitt et al., 2007).

##### Method 2: average distal forearm speed

Hardy et al. (1997) found that ADFS was a good predictor of injury risk across a range of forearm masses during airbag deployment. For smaller forearms, the energy needed to accelerate it to a given speed would impart enough energy to cause a fracture. For a larger, heavier forearm, more energy would be required to accelerate it to the same speed, resulting in a similar injury risk. Hardy et al. (2001) investigated ADFS further using a variety of airbag designs and arm positions, making the results more applicable to other loading conditions.

For ADFS is described by Eq. 49 and shown in Figure 11C. Additional information on calculation of the ADFS can be found in Hardy et al. (2001).

Average distal forearm speed injury risk:

(49) $$p\left(\mathrm{AIS}\geq 2|S\right)=\frac{1}{1+e^{6.749-0.645\cdot S}}$$

where, *S* is the average distal forearm speed.

Using the DAR probabilities of 1% for nominal and 4% for off-nominal (for AIS ≥ 2 injuries), peak distal forearm speeds are 3.3 and 5.5 m/s, respectively.

Because this probability is based on the PMHS population used, mass scaling is necessary to relate the IARVs to the THOR. Hardy et al. (2001) report the linear relationship between forearm mass and ADFS (Eq. 50).

Average distal forearm speed scaling function:

(50) $$\begin{array}{llll} \mathrm{ADFS}\left(\mathrm{scaled}\right) & =\mathrm{ADFS}\left(\mathrm{measured}\right)\; & & \;\\ & \;+1.94\cdot \left(\mathrm{Surrogate}\;\mathrm{Mass}-2.67\right) \end{array}$$

where, ADFS is the average distal forearm speed, Surrogate Mass is the mass of the arm.

Since Eq. 49 needs to be related to the THOR 50th percentile male ATD, the mass of the THOR arm is needed. Since the THOR uses standard Hybrid-III 50th percentile male arms, this information can be found on the Humanetics website (Humanetics Innovative Solutions, 2014). The total weight of the arm assembly is 4.27 ± 0.15 kg. Using Eq. 50, a 50th percentile scaled ADFS injury risk function can be calculated (Eq. 51) as shown in Figure 11D.

Scaled average distal forearm speed injury risk:

(51) $$p\left(\mathrm{AIS}\geq 2|S\right)=\frac{1}{1+e^{9.845-0.645\cdot S}}$$

Using Eq. 51 along with the DAR probabilities, ADFS values for the THOR forearm are 8.1 and 10.3 m/s, respectively. Note that these values are based on elderly PMHS testing with low bone mineral density, causing the values to be conservative for non-deconditioned crewmembers.

##### Method 3: active bracing limit

Instead of defining a value to prevent contact injury to the extremities, another approach is to use active bracing to prevent flail, and thus prevent injury. This method is possible for spacecraft for several reasons: (1) the crew is aware of the impending dynamic loads (aborts may be an exception), the expected loads are low enough that grasping strength should be sufficient to hold the upper extremities in place, and it allows full range of motion of the arms prior to bracing. It should be noted that this method is currently used in military ejection seat testing.

Because an ATD is not designed to simulate active bracing, an alternate method is needed. After consulting with military experts, an approach using break cord is proposed. The break cord would be selected to break at a level expected to be near the maximum grasping strength of a crewmember, thus simulating the force necessary to break a crewmember’s hand away from a handhold. Then if the extremity flails outside of the seat envelop, the design fails. If the break cord remains intact or the extremities stay within the seat envelop, the risk of flail injury is assumed to be mitigated. The values for grasping strength were taken from data compiled by the NASA Anthropometrics and Biomechanics Laboratory, which measures grasping strength on returning crewmembers. These values are reported in the NASA Orion Human-System Requirements Document (National Aeronautics and Space Administration, 2012b). The value chosen, 490 N, is based on the minimum unpressurized suited grasp strength for other operations. This value is similar to the values used in military ejection seat testing.

##### Summary

In comparing the three methods of assessment, the first is least likely to be implementable, since it is based on fifth percentile females and the injury risk function is not useful at very low injury risks.

The second method, however, has great promise for use with the THOR. Additional research is needed to assure that photogrammetry can capture the relevant information necessary to calculate ADFS; otherwise, additional instrumentation would be needed in the forearm. Because the distal speeds reported by Hardy et al. are based on airbag deployment contact, this metric may not be sensitive to the types of flail contact possible in spacecraft; however, the extension of these results to many airbag models and arm positions reported by Hardy et al. give confidence that the metric may be robust.

The third method, is straight-forward and is currently used by other agencies; however, it relies on subjective criteria (whether the arms flail outside of the seat envelop). It is advantageous since it simulates active bracing, which is likely to be the method used to prevent flail.

Based on the three methods described, peak distal forearm speed is chosen as the best option for inclusion in Table 6. Based on the assumptions and extrapolations made to arrive at the specified IARVs, the confidence in these IARVs is rated at a 3. Additional research related to the spaceflight environment, photogrammetry measurement, and low probability of injury is necessary to raise this confidence level.

#### Spaceflight deconditioning factor

To adequately protect the crew upon return from long dwell times in microgravity, a spaceflight deconditioning factor was applied to several of the metrics. These factors were derived from published literature and expert consultation within NASA (Lewandowski et al., 2008). For lower limb injury risk (ankle moments, acetabular force, and contact force), a deconditioning factor of 0.75 was applied. For spinal elements (thoracic spine axial compression, neck axial compression, and neck axial tension), a deconditioning factor of 0.86 was applied.

The assumption is made that returning crew exposed to these forces and moments would have the same risk of injury as non-deconditioned crew exposed to the conditioned IARVs.

### Summary of findings

Table 6 summarizes the IARVs selected based on the existing research. All of the IARVs have been rounded to two significant digits for consistency. Also included are the IARV confidence levels assigned quantitatively to represent the confidence in each metric. Finally, deconditioning factors were applied to several of the metrics to account for spaceflight deconditioning.

## Limitations

Although the stated objective of this study was to investigate new methods for predicting injury from expected spaceflight dynamic loads for all future NASA spacecraft/vehicles, an obvious limitation is that the Orion spacecraft was used as the focal point of discussion in this paper. This results primarily from the authors’ extensive first-hand knowledge and experience with the design of the Orion capsule, as well as the fact that Orion arguably poses the widest array of representative challenges to the consideration and design of future manned spacecraft systems. Observations and determinations with regards to the Orion space capsule specifically – while not necessarily directly transferrable to other systems – are sufficiently characteristic of and retain appropriate fidelity to broadly characterize the achievable range of dynamic loads conceivably experienced by future flight crews in landing and abort sequences.

As can be seen in Table 6, several of the IARVs have low confidence scores. Although preliminary THOR ATD testing has been conducted to assess its performance in the lateral and spinal directions, additional work is needed to develop the associated IARVs. In the event the THOR is found to not be sufficient in these directions, IARV would be developed using the WorldSID ATD.

Several assumptions were made to extrapolate the data from the automotive literature to the spaceflight environment. Most of these assumptions are biased so as to be conservative and were made based on the best available data to date. These assumptions include:

- The loading rates used in the automotive literature are good approximations of the loading rates expected in the spacecraft.
- The THOR ATD will be biofidelic at the specified loading rates.
- Extrapolations of injury from PMHS are appropriate.
- Extrapolations from other ATDs and models are appropriate.

Because the 50th percentile male THOR ATD is the only size available, it is not representative of the entire astronaut anthropometric range. Those crewmembers on the two ends of the spectrum, the 1st percentile female and the 99th percentile male, may have different injury risk than the 50th percentile male. Furthermore, there is no way to simulate, or physically test, other sizes because only the 50th percentile male is available. The current NASA OP requirements state that occupants may range from 1st percentile female to 99th percentile male. In addition, gender and age may contribute to injury risk. Microgravity affects the musculoskeletal system, which has an unknown effect on a crewmember’s impact tolerance. Currently, appropriate injury risk functions that account for these effects are not available about these effects. Additional research is again needed to address this gap in knowledge. The pressure suit adds another level of uncertainty that will require a better understanding of its contribution to injury risk. The IARVs in this report do not completely account for these risk factors, so it is unclear if the proposed limits will be protective for all crewmembers, or if adjustments will be required to the THOR ATD IARVs to account for these variables. Additional research is needed to address these factors.

Finally, although the THOR is an advanced ATD, it is not a perfect human surrogate, and does not respond in a way that exactly mimics the human response. The injury risk may be accurate for a range of probabilities and seated environments, but may deviate for responses outside of the region where the test data were obtained.

## Conclusion and Recommendations

There exist unique challenges when attempting to determine reliable and achievable protective mechanisms and procedures for spaceflight through the application of non-spaceflight derived injury biomechanics considerations to the design of future spacecraft. The purpose of this paper is to discuss the pathways toward developing new and improved methodologies for predicting injury from expected dynamic spaceflight loading environments through the exploitation of a broad range of extant scientific thought, research, development, and data from various injury biomechanics sources and venues. The authors seek to illuminate a proposed pathway to couple existing knowledge with future, directed research aimed at improving upon current metrics and achieving a better understanding of the inherent and unique challenges related to developing accurate and robust injury prediction, prevention, and mitigation tools for the spaceflight domain.

As discussed in this paper, there are numerous reasons why it is not appropriate to directly apply IARVs derived from other industries – such as the automotive field – to the spaceflight domain. Through a process of carefully considering the context and range of spaceflight design considerations, a thorough evaluation of dynamic loading parameters with the highest potential of resultant injuries most critical to mission success, and long-term crewmember health, an understanding of considerations of crew member deconditioning, suit related factors – such as placement and design of rigid elements, mission critical components and restraint systems, factors related to astronaut gender and anthropometrics, as well as an comprehensive assessment of acceptable NASA injury risk posture and criteria – the NASA OP team has identified the THOR ATD as the most appropriate test device for NASA’s future use in the development of improved injury protection standards.

This recommendation is made with the knowledge and caveat that the test device and its attendant mathematical models are specific to the injury and risk assessments detailed and described in the sections above. Furthermore, it should be noted that the general approach taken toward injury protection assessment seeks to avoid and/or mitigate injury at a “lowest possible” level within the loading rate parameters expected in “nominal” or “near-nominal” conditions. Such limitation of instantaneous dynamic forces (e.g., acceleration) in the aforementioned space flight phases of interest (i.e., launch abort and landing) to “acceptable” levels, thereby, presumably precludes the risk of fatal injury. Therefore, the NASA team has begun the deliberate process of developing a set of preliminary IARV’s – in full collaboration with both NASA and industry subject matter experts and multi-disciplinary review panels – as a starting point for the eventual adoption of improved crew protection standards and policies.

Specifically, a NASA convened “Occupant Protection” team expert summit primarily for utilization in the Orion capsule project, but, as well, to speak to the needs of future commercial space crew vehicles. A multi-disciplinary team of experts defined a five phase plan to address issues from current standards to anthropometric test device capabilities/selection and FEM issues, to human subject testing and correlation to test device data, to the development of injury risk functions and future NASA protection standards. The expert panel further concluded that NASA should develop a list of “critical injuries” that require mitigation and which would drive future protection and design requirements for vehicles and support/egress/rescue systems alike.

The expert panel’s deliberation and resulting recommendations provided input to the development of Table 3 through Table 6 and encompass both the methodology and associated metrics, which were used to determine the “best of breed” qualities of existing and future anthropometric test device capabilities validated against the previously described NASA critical injury list. This process resulted in the expert panel determining that the THOR ATD as the best overall choice for future NASA development (in collaboration with industry, academia, and other governmental stakeholders), injury prediction, analysis, and IARV determination. Whereas the THOR ATD has its inherent limitations, both the panel and the authors of this paper recommend its adoption for future work in this area.

Finally, the information contained in this report is intended to provide a first step toward redefining NASA Human Spaceflight Standards for injury biomechanics. The IARVs specified are currently a best estimate of acceptable and “safe” risk values. Given the acknowledged limitations of this work, additional efforts aimed at optimizing knowledge by further leveraging data mining techniques and emphasizing validation with human volunteer testing is highly recommended. Once such data is collected and analyzed it would be desired – and advisable – to further examine and potentially revise these IARVs with their possible incorporation into NASA’s Human Spaceflight Standard, its human-systems integration requirements (HSIR) Document, and the ISS Crew Transportation and Services Requirements (National Aeronautics and Space Administration, 2011, 2012a,b). Concurrently, these future improved specifications may be used to complement, and/or possibly supersede, the presently utilized Brinkley dynamic response criteria.

## Author Contributions

Jeffrey T. Somers was involved in all aspects of the research and worked on development of the injury risk functions. Nathaniel Newby and Shean Phelps assisted with the development of the injury risk functions and overall technical content. Charles Lawrence, Richard DeWeese, and David Moorcroft assisted with the selection of the ATD and ATD metrics.

## Conflict of Interest Statement

The authors declare that the research was conducted in the absence of any commercial or financial relationships that could be construed as a potential conflict of interest.

## Abbreviations

AIS, abbreviated injury scale; ATD, anthropomorphic test device; BDRC, Brinkley dynamic response criterion; BrIC, brain rotational injury criteria; CAMI, Civil Aeromedical Research Institute; CG, center of gravity; CM, command (crew) module; CSDM, cumulative strain damage measure; DAR, definition of acceptable risk; EAFB, Edwards Air Force Base; EVA, extravehicular activity; FAA, Federal Aviation Administration; FE, finite element; FEM, finite element modeling; FTSS, First Technology Safety Systems; GRC, Glenn Research Center; HIC, head injury criteria; HRP, Human Research Program; HSIR, human-systems integration requirements; IARVs, injury assessment reference values; IRL, Indy Racing League (aka: INDYCAR™); ISS, international space station; JARI, Japan Automobile Research Institute; JSC, Johnson Space Center; KSC, Kennedy Space Center; LEO, low earth orbit; LM, Lockheed Martin; LOC, loss of consciousness; MPCV, multi-purpose crew vehicle; mTBI, mild traumatic brain injury; NASA, National Aeronautics and Space Administration; NBDL, Naval Biodynamics Laboratory; NESC, NASA Engineering and Safety Center; NHTSA, National Highway Traffic Safety Administration; OP, occupant protection; PMHS, post-mortem human surrogates; SID, side-impact dummy; SLS, space launch system; SRB, solid rocket boosters; TBI, traumatic brain injury; TBD, to be determined; THOR, test device for human occupant restraint modification kit; USAARL, United States Army Aeromedical Research Laboratory; USSR, United Soviet Socialist Republic; WSTF, White Sands Test Facility.
